# Trust me, I’m a researcher!: The role of trust in biomedical research

**DOI:** 10.1007/s11019-016-9721-6

**Published:** 2016-09-15

**Authors:** Angeliki Kerasidou

**Affiliations:** The Ethox Centre, Nuffield Department of Population Health, University of Oxford, Oxford, UK

**Keywords:** Trust, Reliance, Trustworthiness, Biomedical research, Research ethics, Professional integrity

## Abstract

In biomedical research lack of trust is seen as a great threat that can severely jeopardise the whole biomedical research enterprise. Practices, such as informed consent, and also the administrative and regulatory oversight of research in the form of research ethics committees and Institutional Review Boards, are established to ensure the protection of future research subjects and, at the same time, restore public trust in biomedical research. Empirical research also testifies to the role of trust as one of the decisive factors in research participation and lack of trust as a barrier for consenting to research. However, what is often missing is a clear definition of trust. This paper seeks to address this gap. It starts with a conceptual analysis of the term trust. It compares trust with two other related terms, those of reliance and trustworthiness, and offers a defence of Baier’s attribute of ‘good will’ a basic characteristic of trust. It, then, proceeds to consider trust in the context of biomedical research by examining two questions: First, is trust necessary in biomedical research?; and second, do increases in regulatory oversight of biomedical research also increase trust in the field? This paper argues that regulatory oversight is important for increasing reliance in biomedical research, but it does not improve trust, which remains important for biomedical research. It finishes by pointing at professional integrity as a way of promoting trust and trustworthiness in this field.

## Introduction

In biomedical research lack of trust is seen as a great threat that can severely jeopardise the whole biomedical research enterprise (Kass et al. 1996; Mastroianni 2008). Practices such as informed consent and also the introduction of administrative and regulatory oversight of research in the form of research ethics committees (RECs) and Institutional Review Boards (IRBs), are to ensure the protection of future research subjects and, at the same time, restore public trust in biomedical research (Pettit 1992; Bok 1995; Yarborough and Sharp 2002; Faden et al. 2005; Dixon-Woods and Ashcroft 2008). Empirical research also testifies to the role of trust as one of the decisive factors in research participation and lack of trust as a barrier for consenting to research (Sugarman et al. 1998; Corbie-Smith et al. 1999; de Melo-Martín and Ho 2008; Marsh et al. 2008; Slegers et al. 2015).

O’Neill, on the other hand, maintains that reports of loss of trust might be misleading, as they do not reflect the way people actually behave (O’Neill 2002a, b). If people *really* distrusted biomedical research, she argues, then a decrease in biomedical research involving humans should be the outcome of this, rather than the increase that is observed (O’Neill 2002a, b). This seems to imply that trust is rather unimportant, or at least not as important in biomedical research as is usually led to be believed. If this is true, researchers could stop worrying about building trust relationships with their research participants and participating communities, and just get on with their research. As long as they follow the appropriate rules and regulations, whether they are trusted by their participants and participating communities or not, should not have an effect on their research.

Some might recoil at the idea that building trust relationships should be scrapped from the ‘ethical’ requirements of research. For example, community engagement has been promoted as the most effective way of building trust relationships with participants and communities, and for this reason, is increasingly seen as essential requirement of ethical research (Marshall and Rotimi 2001). Equally, the requirement for consent has also been defended as a method for trust building (Faden et al. 2005). What is often missing from such accounts, however, is a clear definition of trust. Trust, like dignity and respect, has become yet another nebulous term in research ethics which is often invoked but rarely examined in this context.

This paper is coming to address this gap. It starts with a conceptual analysis of the term trust. It compares trust with two other related terms, these of reliance and trustworthiness, and offers a defence of Baier’s attribute of ‘good will’ as a basic characteristic of trust. It then, proceeds to consider trust in the context of biomedical research by examining two questions: First, is trust necessary in biomedical research?; and second, do increases in regulatory oversight of biomedical research also increase trust in the field? It argues that regulatory oversight is important for increasing reliance in biomedical research, but it does not improve trust. The paper argues that trust is, however, important for biomedical research, and it finishes by pointing at professional integrity as a way of promoting trust and trustworthiness in this field.

## Trust, reliance and trustworthiness

Trust can take different forms depending on the person on whom we declare our trust, and also on the situation. There are people that we trust wholeheartedly and perfectly, for example our mother or spouse, and others that we trust with something specific or only in a particular situation, for example our business-partner *with* the running of our company (but not with looking after our children). Trust developed between two individuals is called personal trust, whereas trust directed to institutions, professional bodies, companies, governments or other large social systems is referred to as institutional, a personal or impersonal trust. Some question institutional or apersonal trust arguing that trust is a relationship developed overtime between two individual agents (Mayer et al. 1995; Rousseau et al. 1998). It could be argued, however, that in so far as institutions, companies and other large social systems can make decisions and act as one body, they have a unique moral character—which is different from and cannot be reduced to the character of its members—and therefore can be judged and trusted as distinct moral agents. In this paper, the nouns trustors and trustees would refer to both individuals and institutions, unless differently specified.

Irrespective of the different forms trust can take, all such relationships share some common characteristics: (1) assumption of ‘participant stance’ or vulnerability; (2) attitude of ‘good will’ towards the trustor; and (3) voluntariness. Assuming a ‘participant stance’ (Holton 1994; Wright 2010), means that the trustor enters a relationship where she believes that the trustee can decisively influence the outcome of the entrusted action (Wright 2010, 618f). In other words, the trustor knowingly makes herself vulnerable towards the trustee. The adoption of this stance is what justifies feelings of gratitude, when trust is confirmed, or betrayal, when trust is disproved. I trust my friend not to reveal a very important secret that I entrusted in her. If she honours my trust and keeps my secret, I do not only feel assured but also grateful for her behaviour. If she does, however, reveal my secret to a third party, then I feel not only upset about it, but also betrayed by her. As both Holton (1994) and Wright (2010) point out, these feelings of betrayal or gratitude are appropriate because we *trust* our friends, we do not simply rely on them.

In order for beliefs of betrayal or gratitude to be justified, and for the belief that the trustee can influence the outcome of the action to be reasonable, the trustee has to be aware that trust has been placed on them. Their awareness of entrustment adds another component in their decision making. If one knows that their friend trusts them with certain information, this knowledge will influence their behaviour and decision making. To return to the earlier example, the friend will not only need to consider whether the information she has should be revealed or not, but also the effect her decision will have upon her friend, the trustor. This leads us to the second characteristic of trust relationship, which is the assumption or belief that the trustee has ‘good will’ towards the trustor (Baier 1986). Holton and Wright object to the role of good will as a necessary component of a trust relationship. One can imagine a trust case where good will is not required, they maintain, for example when an army decides to raise the white flag and trusts that their adversaries will not kill them (Holton 1994; Wright 2010). O’Neill also rejects the necessity of feelings of good will in trust relationships. We all take medication, she argues, that has been developed by pharmaceutical companies for which we have no reason to assume they have good will towards us (O’Neill 2002a, b). A respond to this criticism lies in the distinction between trusting behaviours—acting like we trust- and trusting attitude—actually trusting (Hall et al. 2001). An army surrendering to their opponent when there is *no other option* available, other than certain death, can hardly be described as an act of trust.

Entering a relationship, in which we assume a participant stance, out of mere necessity rather than choice cannot be described as trust relationship.^1^ Voluntariness, therefore, is the third characteristic of trust relationships. Trust cannot be demanded and it is only freely given. If no options are available or one is presented with an ‘empty choice’ (Kingori 2015), then the action is not motivated by trust. Similarly, continuing to use medicines produced by pharmaceutical companies does not necessarily denote a relationship of trust. People expect that pharmaceutical companies will not poison or harm them with their products, not (necessarily) because they feel that the pharmaceutical industry has good will towards them, but because they (also) believe that the system that regulates the pharmaceutical industry is been developed within a framework that seeks and supports the welfare of the people, and therefore has good will towards society. So, what appears as an act of trust towards the pharmaceutical industry is actually an act of trust towards the overarching system that wills our good. What these characteristics of trust -vulnerability, voluntariness and assumption of good will-reveal is the moral component and underpinnings of trust. We can say that we trust someone when we know that his actions could make us happy (feelings of gratitude) or hurt us (feelings of betrayal),^2^ and we expect that our vulnerability towards them would be taken into account ‘directly and favourably’ (Jones 1996) when they consider how to act. We do not trust mere agents, but rather we trust *moral agents*. We expect those whom we trust to behave not just as we assume they will, but as we believe they should or ought to (Walker 2006; Jones 2012).

Reliance, being able to rely on someone to act in a particular way, is often used as a synonym to trust. However, there is a clear distinction between these two terms. Reliance does not justify feelings of gratitude or betrayal, nor does it necessitate ‘good will’ between the partners (Jones 1996). This means that although we can be harmed or wronged by an individual or an institution on whom we relied but they failed us, we cannot be hurt by them –at least not in the same way that breaking trust hurts. We rely on the supply of gas to our house to use the gas cooker, but we do not assume malevolent intentions to our gas supplier, if the supply gets interrupted whilst we are cooking our dinner. When we deposit a check into our account we rely on the bank clerk in our local branch to process the payment. If he fails to do so and our money goes missing we will feel annoyed, upset, even angry and will use all legal routes available to retrieve our money, but we will not feel betrayed by him; at least not as one would, if we were to find out that our best friend had been stealing money from us. Reliance is an act of dependence based on the likely prediction of the other’s behaviour. It does require or entail the assumption of good will, nor the expectation that our vulnerability will be considered directly and favourably (Baier 1986; Jones 1996).

Another concept that requires some clarification in relation to trust is that of trustworthiness. Trustworthiness relates to the person who is being trusted. It refers to the exhibit of characteristics of the trustee, which indicate that the trustee has good will towards the trustor. A person can be characterised as trustworthy when she ‘acknowledges the value of the trust that is invested in [her, and] uses that to help [her] rationally decide how to act’(Wright 2010, 622). As O’Neill points out, building and restoring trust relationships, in effect means building or restoring individuals’ and institutions’ trustworthiness. Given that trust is something that is voluntary given and cannot be demanded, the only way of restoring trust is by enhancing trustworthiness and thus, creating the conditions for trust relationships to ensue and flourish (O’Neill 2002a, b).

## Trust in the context of biomedical research

A trust relationship in biomedical research can take any of the aforementioned forms. It can be a case of personal trust between two individuals (e.g. a researcher and research participants) or it can be a case of apersonal trust between an individual and an institution (e.g. research participant and research institutions) or between two institutions (e.g. two collaborating research institutions).

The long list of scandals in biomedical research, from the Nazi doctors’ experiments to the more recent cases of Jesse Gellsinger (Stolberg 1999), the SUPPORT neonatal trial (Lantos 2014) and the Macchiarini case (KI 2016), it has been argued, has undermined public trust in it. The introduction of laws and rules to regulate biomedical research, and the promotion of transparency and accountability has been seen as the appropriate response to the loss of trust and as a way to reinstate and promote trustworthiness (Bosk 2002). Building on the Nuremberg Code of 1948, the Declaration of Helsinki first published in 1964 by the World Medical Association,^3^ and the Belmont Report in 1979 stipulated the ethical parameters of biomedical research involving human subjects. Institutional Review Boards (IRBs) and, Research Ethics Committees (RECs) were established by research and medical institutions to oversee biomedical research, and to ensure researchers’ compliance with research ethics regulations and guidelines, and to safeguard the participants’ welfare (Ellis 1999; EC 2001, The medicines for human use (clinical trials) regulations 2004). Consent forms are now obligatory for all biomedical research that requires the participation of human subjects. It is increasingly the norm for the transfer of research materials between researchers and institutions to be regulated by material transfer agreements (MTAs). Also, all clinical trials are now registered on an online open access database (WHO ICTRP). Two questions should be examined in this context. First, whether increases in regulatory oversight of biomedical research also increases trust in the field or just reliance and compliance; and second, whether trust is, indeed, necessary in biomedical research

## Regulatory oversight and trust in biomedical research

The common response to the loss of trust in a profession or institution is the introduction of new agreements, the strengthening of regulations and accountability pathways (Pettit 1992), and the establishment of ‘guardians of trust’ (Shapiro 1987). In biomedical research this response took the form of national and international RECs and IRBs, MTAs, international clinical trial registries and a plethora of rules of conduct and guidelines for researchers and institutions. Some authors have suggested that trustworthiness can be increased by such methods, for example by introducing sanctions, which ensure that the person or institution on whom trust has been placed will act as expected (Hardin 1996). Others, on the other hand, argue that regulations and sanctions do not increase trustworthiness but, rather, compliance and reliance (Wright 2010). This is because the reason the person has decided to act in a particular way is self-interest, rather than the desire not to betray and hurt someone. Mouzas et al., describe reliance as the rational manifestation of a consent-based exchange where ‘contracting parties […] bring to the exchange certain entitlement and they manifest their consent to the transfer of these entitlements’(Mouzas et al. 2007, 1021). Methods that increase accountability and compliance can positively affect relationships of reliance by increasing commitment and introducing or strengthening self-interest reasons to behave a certain way. Through this process relationships between partners are strengthened.

In trust relationships, though, what matters is not only whether the trustee is motivated to fulfil the entrusted act, but also the reasons that motivate him or her. Trust is a relationship where the trustor becomes vulnerable to the trustee by recognising and accepting the effect the trustee’s decision would have on the outcome of the entrusted action, and hopes that the trustee will honour the relationship and fulfil the entrusted act. The vulnerability of the trustor and a genuine concern and consideration for him or her, namely an attitude of good will towards them, are the motivating factors of a trust relationship. Persons or institutions that wish to become more trustworthy, as oppose to only appear to behave more trustworthily, could achieve this by the adoption of an attitude of good will towards the trustor; that is by acknowledging the trusting party’s vulnerability and taking this into direct and favourable consideration. The difference between reliance and trust lies on whether the motivational reason behind the action is good will or self-interest.

Introducing sanctions and placing safeguards to ensure compliance and increase reliance could be seen as the opposite of trust, as an action of distrust (O’Neill 2002a, b). When we ask our friend to pick up the post for us, we do not feel the need to threaten them with sanctions in case they fail do to so.^4^ We trust them that they will not ‘let us down’. If we were to present them with a contract that said that in the case they failed to pick up our post, they will incur a penalty of £20, this would mean that we do not trust them and we are trying to protect ourselves from being ‘let down’ by attempting to promote their reliability. The introduction of rules and regulations can be seen as a confirmation of the untrustworthiness of a party, as it indicates that this party is not motivated by good will to act trustworthily, and that their behaviour needs to be guided and controlled by rules, contracts, regulations and penalties. Shapiro calls this the ‘paradox of trust’. She observes that ‘[b]y buying or requiring “fidelity” insurance, we discourage internal discipline and control and thereby increase the likelihood that trust will be abused’ (Shapiro 1987). Similarly, Pettit expressed his reservations and scepticism regarding the introduction of RECs and IRBs, which, he feared, would corrupt professional integrity and lead to more unethical behaviour (Pettit 1992). What Shapiro and Pettit imply is that there is a reverse relationship between regulations and trust. Ensuring reliability adversely affects trust and trustworthiness. It would seem appropriate to suggest that in order to promote trust and trustworthiness in biomedical research, it is necessary to decrease the level of oversight and withdraw some of the rules and regulations that govern it.

## Is trust necessary in biomedical research?

Before we start dismantling ethics committees and scrapping regulations, however, it is important to consider whether trust is actually relevant or even necessary in biomedical research. If one can rely on a researcher or research institution that they will act as expected, is it also necessary that one should be able to trust them, as well? If by revising and strengthening rules and sanctions all parties’ compliance can be ensured, it can only be a good thing that researchers, participants, research institutions, public and private do not *have to* trust each other anymore. For in order for trust to still be relevant in biomedical research, it needs to be demonstrated that one of the parties assumes a participant stance, namely it assumes a vulnerable position and opens itself up to be validated or betrayed by the other party.

The relationship between researchers, their institutions and research participants is often described as a consent-based relationship, which depends on reasonable expectations and proven capacity. Strict regulations, greater transparency, clear system of accountability make the partners or participants feel that they can rely on the institution or researcher to do what it has been agreed. If biomedical research can be described *solely* as a consent-based exchange, then, it can be argued, the existence of trust, or lack thereof, between the stakeholders is irrelevant. In cases where reliability is low and accountability is lacking, rules and regulations can be introduced to make the exchange between the two parties more equal and fair; reliance can be promoted through structures and guidelines that increase accountability. What increased reliance can achieve, is to bring the collaborating parties together as equals (or near equals) and thus, mitigate power imbalances between them. Once reliance and compliance is secured, more successful collaboration will ensue, as both partners, being two institutions, institutions and research participants, or researcher and participant, will feel confident and secure in their relationships (Yarborough et al. 2009).

Increasing compliance is undeniably important, but, as mentioned above, it cannot solve the trust problem in biomedical research. It is worth probing a bit further the question of why people still remain sceptical towards biomedical research even though all aspects of it, from the recruitment of participants, to sample and data collection, sharing and use is closely regulated. Do research participants make themselves vulnerable (i.e. assume a participant stance) by participating in research, and if yes, how?

All research participation entails some level of risk to the participant. Consent forms are a necessary requirement, as they lay down the terms and conditions of the relationship. They inform prospective participants of the specific risks, potential benefits (e.g. post-trial access to medication at an individual or community level), what is required from participants and what can be expected from researchers and their institutions, and allow individuals (and/or communities) to make a free and informed decision regarding their participation. However, informing potential participants openly and transparently about the risks of research does not absolve researchers from the obligation to take any possible steps to minimize potential risks, to provide safeguards for the participants’ welfare and to ensure fair distribution of research benefits. For participants to actually trust the researchers, it means that they believe that the researchers have designed and will conduct the research with an attitude of good will towards them. As O’Neill observes, the signing of consent forms does not confirm trust, rather it presupposes it (O’Neill 2002a, b).

Furthermore, by becoming research subjects, all participants ‘surrender’ their health and health related information (in various degrees, depending on the type of biomedical research) to the hands of research professionals and institutions. Drawing a parallel with the doctor-patient relationship here might be helpful to clarify this point, although it is important to note that researchers do not have the same obligations towards the participants, as doctors do towards their patients (Rhodes 2005). Patients trust doctors not only because there is a clear legislation and a system of accountability in place to protect them, but also because they feel that the doctor has a good will towards them. The patient invariably assumes a participant stance towards his or her doctor. This means that the patient accepts that the doctor’s actions would have a definitive effect on the outcome of the entrusted action—restoration of health. A clear and effective system of accountability notwithstanding, the patient is always in a vulnerable position. Competency and concern about patients’ interests suffices to justify a doctor’s reliability. Trust however, requires that the doctor is responsive to the patient’s vulnerability, and that she or he takes it into account when considering his or her course of action.

Similarly, in biomedical research participants can risk their health by participating in drug trials or put themselves into emotional, social or economic risk by participating in research that could lead to ethnic or group stigmatization (de Vries et al. 2012). Again, systems of control and accountability cannot compensate for putting oneself in a position of vulnerability. The participants knowingly assume a participant stance towards the researchers, and they need to feel that researchers and research institutions have a good will towards them and towards society as a whole (Molyneux et al. 2005; Tindana et al. 2011). An attitude of good will in the research context would mean that researchers would acknowledge the participants’ vulnerability and take it into account when considering how to design, conduct and implement their research.^5^

Even in research where participants are healthy volunteers and there is minimal physical risk involved, such as in genomic research, participants also need to assume that the researchers and institutions which will use and curate their genetic information have a good will towards them (Hansson 2005; Tindana et al. 2012).^6^ Most genetic and genomic data nowadays are collected under broad consent, meaning that the participants are not informed about the particular future uses of their genetic information, or about who might be given access to them. The participants are asked to trust ethics committees, researchers, biobanks and universities that their genetic information will be put to good use and that, directly or indirectly, their interests and welfare will be promoted. The rules of this agreement are open and vague and the participants invariably place themselves into a position of vulnerability by entering a relationship where the trustee can decisively influence the outcome of the entrusted action. Participants have the option to choose which institution to trust, the same way that people choose which person to befriend. They can chose to donate their biological and genetic information to institutions that they believe, have good will towards them, institutions that will take into account their situation and needs before they decide how to act. Namely, they can choose to participate and collaborate only with researchers and research institutions that they deem to be trustworthy.

## Implications for biomedical research: the importance of professional integrity

So far, this paper has drawn a distinction between trust and reliance and have argued that both types of relationship, that of reliance and of trust, are appropriate and relevant for biomedical research. Also, it has discussed ways that can promote reliance. These include the introduction of rules and regulations, contracts and clear systems of accountability. Ways to promote trust, however, can be more difficult to identify. Reliance is a calculable relationship that depends on a rational and mutual enforceable agreement that serves the interests of both parties. Trust is an emotive relationship of dependency^7^ associated with risk and vulnerability. Trust cannot be enforced and it greatly depends on the character of both the trustor and the trustee.

It is possible for researchers and research institutions to develop a character that can promote trust relationships by showing their good will towards individuals and the public as a whole. When we talk about character within a profession, we mainly refer to professional integrity. Professional integrity is linked with the identity to which professionals subscribe (Miller et al. 1998). Virtues such as courage, respectfulness, responsibility, humility and prudence can be associated with the character of the good biomedical researcher (Macfarlane 2010). These professional characteristics or virtues cannot be prescribed nor can be they enforced by rules and regulations, but develop through a process of education and habituation. Rules and regulations are important in setting the scene of biomedical research, but ‘more should be expected from scientists when it comes to responsible conduct of research’ than just conforming to rules and regulations (Institute of Medicine 2002). By appealing to the conscience of individual scientists, the scientific community as a whole should seek to evoke the highest possible standard of research behaviour’ (Institute of Medicine 2002). Appropriately designed educational programs that raise awareness on the ethical issues of biomedical research, and promote ethical sensitivity and ethical reasoning have been suggested as a way to promote professional integrity (Miller et al. 1998; Johnsson et al. 2014).

The notion of professional integrity does not only apply to individuals but also to institutions. Institutions are not just the amalgamation of the individuals that constitute them, but they also form a distinct entity that can express its own moral character thought its collective actions. Characteristics or virtues such as fairness, openness, transparency, consistency, and also dedication to ethics and ethical research can be seen as indications of an institutions’ moral character and promote trustworthiness (Baier 2004). ‘For institutions, it is a matter of creating an environment that promotes responsible conduct by embracing standards of excellence, trustworthiness, and lawfulness that inform institutional practices.’(2002) Particularly, demonstrating great interest in promoting ethical research, for example by incorporating a dedicated ethics team into their system, could play an important role in promoting professional integrity and consequently, public trust. By giving appropriate attention to the ethical component of research and by dedicating resources for the investigation and analysis of ethical issues, it would communicate to the potential partners and participants that ethics is not just a formality but a core component of their professional character (Kerasidou and Parker 2014). But most importantly, institutions should openly and demonstrably support and uphold their social role. The social role of biomedical research is the generation of new knowledge and the discovery of effective therapeutics with the ultimate aim to improve the health and welfare of all people. Research institutions should commit themselves to fulfilling this aim. An institution’s actions, past and present, their track record in achieving their social mission and their other relations and affiliations will be the indicators of its moral character. Participants and collaborators would be able to place their trust to such an institution, as they will have good reasons to believe that it will honour their vulnerability and will assume a position of good will towards them.

## Conclusion

Establishing a regulatory framework that governs and regulates biomedical research has increased its reliability but has had very little effect in making it more trustworthy. To the contrary, it has been argued that promoting reliability can have an adverse effect on trust. Yet, a trustor-trustee relationship is important and relevant in biomedical research as vulnerability and belief in the trustee’s good will form the basis of that relationship. If researchers and research institutions want to restore trust in biomedical research, they should focus not only on promoting their reliability through regulatory and compliance systems, but also on promoting their trustworthiness. This can be achieved by fostering and encouraging ethical conduct and ethical research through educational programs, dedicated ethics teams and most importantly engagement with stakeholders to help promote the social value of research.

## Footnotes

1 An exception to this rule would be the infant-mother relationship, where the newborn baby seems to ‘trust’ the mother to care for it and not poison it with her milk. Two things can be said as a response to this counterargument. Firstly, I believe that the infant-mother case says more about the importance of good will in trust relationships rather than the role of necessity. It is safe to assume an innate urge to turn to the being that gave birth to us for safety and nutrition. It is when this basic urge is not fulfilled, when we realise that the person in question does not have our good will at heart that feelings of distrust emerge. Secondly, I would draw a distinction between a trust relationship and *the appearance of* a trust relationship to an external observer. Relationships of necessity might have the appearance of a trust relationship to an external observer who cannot test or verify the trustor’s voluntariness and belief in the trustee’s good will. Yet the mere appearance of something as *y* does not justify it actually being *y*. For an interesting analysis of the mother-infant relationships see: Baier (1986).

2 When someone decides to betray our trust, this does not necessarily mean that s/he wills our ill too. Consider this example: Person A is diabetic and is at the hospital for dialysis. Person B visits person A. Person A reveals to person B that he has a stack of sweets and chocolates under his pillow, but vows person B not to say anything to his doctors because they will take the sweets away from him. Person B being aware of the seriousness of person’s A health condition decided to break his friend’s trust and tell his doctors about the hidden sweets. One can contest whether person B promising not to tell the doctors’ about person’s A sweets was the right thing to do, but still one can accept that person B reveals the secret out of good will towards his friend.

3 Since 1964 the Helsinki Declaration has been revised many times, always trying to safeguard research participants and consequently increase researchers’ accountability towards participant’s welfare. The latest revision was in 2013.

4 The loss of trust that could result from being ‘let down’ repeatedly, and unjustifiably, does not count as a sanction.

5 It should be made clear that an attitude of good will towards participants does not require researchers to always act at the participants’ best interests. Indeed, often researchers would invite volunteers to participate in risky research that could potentially put their health and even life into danger (e.g. first-in-human clinical trials). It does, however, mean that researchers would consider the participants’ vulnerability and their counting on them as a substantive reason for minimising risk and introducing appropriate steps and procedures avoid harming participants; rather than, for example, considering consent as the sole justification for all kinds and types of risk. (I thank one of the anonymous reviewers for this point).

6 Of course, not all biomedical research is equally risky, nor all research participants expect direct and individual benefits from participating. A great majority of research participants decide to become research subjects not for their own good but for the good of others and of society in general. They are primarily motivated by altruism rather than personal gain, and they expect that researchers and research institutions share the same motivations, namely that they are guided by a desire to serve the social good rather than a selfish motivation of personal gain. This is the reason why people are more inclined to participate in research conducted by researchers and institution they perceive have more of a social character, e.g. universities, rather than private institutions e.g. pharmaceutical companies. See: Pullman et al. (2012).

7 This is not to say that it is not rational.
